# Supplementary material for: Saccharomyces boulardii (CNCM I-745) improves intestinal damage in sepsis by remodeling intestinal flora structure
Source: Front Cell Infect Microbiol. 2025 Jul 31;15:1602792. doi: 10.3389/fcimb.2025.1602792 (PMC12350329; doi:10.3389/fcimb.2025.1602792)
Supplement: Supplementary file 1 [file DataSheet1.zip › summary/summary/8_Advanced_analyse/2_PICRUSt2_result/KEGG/Group.DW4_vs_DW0/PICRUSt2_KEGG_level2_Group.DW4_vs_DW0_diff.pdf]

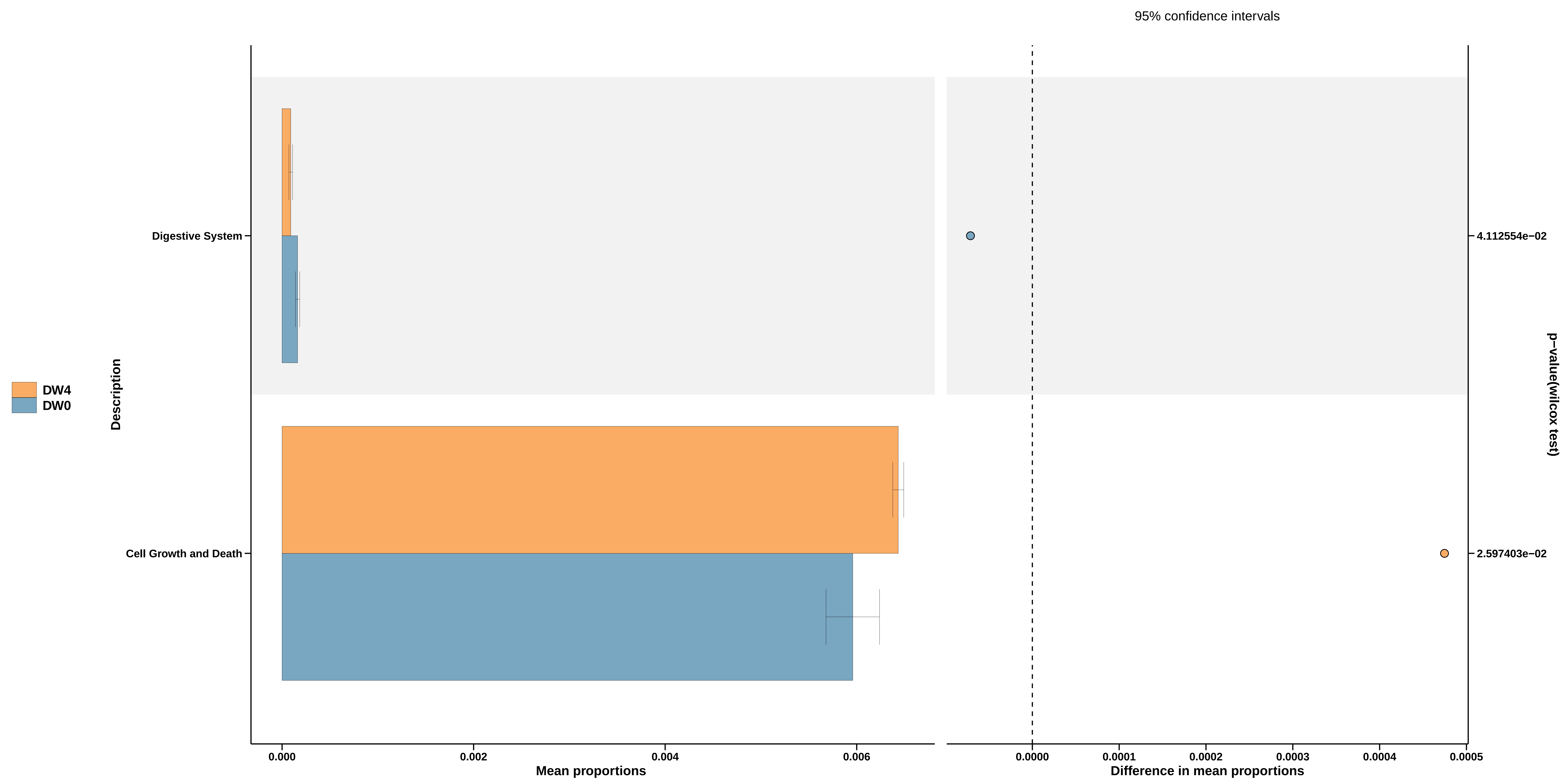

95% confidence intervals
DW4
DW0
Description
Digestive System
Cell Growth and Death
0.000
0.002
0.004
0.006
Mean proportions
0.0000
0.0001
0.0002
0.0003
0.0004
0.0005
Difference in mean proportions
4.112554e−02
2.597403e−02
p−value(wilcox test)
